# Platelet-Rich Plasma: The Choice of Activation Method Affects the Release of Bioactive Molecules

**DOI:** 10.1155/2016/6591717

**Published:** 2016-09-08

**Authors:** Carola Cavallo, Alice Roffi, Brunella Grigolo, Erminia Mariani, Loredana Pratelli, Giulia Merli, Elizaveta Kon, Maurilio Marcacci, Giuseppe Filardo

**Affiliations:** ^1^Laboratory RAMSES, Rizzoli Orthopaedic Institute, Via di Barbiano 1/10, 40136 Bologna, Italy; ^2^Nano-Biotechnology Laboratory, Rizzoli Orthopaedic Institute, Via di Barbiano 1/10, 40136 Bologna, Italy; ^3^Laboratory of Immunorheumatology and Tissue Regeneration, Rizzoli Orthopaedic Institute, Via di Barbiano 1/10, 40136 Bologna, Italy; ^4^Department of Medical and Surgical Science, University of Bologna, Via Giuseppe Massarenti 9, 40138 Bologna, Italy; ^5^Clinical Pathology Unit, Rizzoli Orthopaedic Institute, Via di Barbiano 1/10, 40136 Bologna, Italy; ^6^I Orthopaedic and Traumatologic Clinic, Rizzoli Orthopaedic Institute, Via Pupilli 1, 40136 Bologna, Italy; ^7^Biomechanics Laboratory, Rizzoli Orthopaedic Institute, Via di Barbiano 1/10, 40136 Bologna, Italy

## Abstract

Platelet-Rich Plasma (PRP) is a low-cost procedure to deliver high concentrations of autologous growth factors (GFs). Platelet activation is a crucial step that might influence the availability of bioactive molecules and therefore tissue healing. Activation of PRP from ten voluntary healthy males was performed by adding 10% of CaCl_2_, 10% of autologous thrombin, 10% of a mixture of CaCl_2_ + thrombin, and 10% of collagen type I. Blood derivatives were incubated for 15 and 30 minutes and 1, 2, and 24 hours and samples were evaluated for the release of VEGF, TGF-*β*1, PDGF-AB, IL-1*β*, and TNF-*α*. PRP activated with CaCl_2_, thrombin, and CaCl_2_/thrombin formed clots detected from the 15-minute evaluation, whereas in collagen-type-I-activated samples no clot formation was noticed. Collagen type I produced an overall lower GF release. Thrombin, CaCl_2_/thrombin, and collagen type I activated PRPs showed an immediate release of PDGF and TGF-*β*_1_ that remained stable over time, whereas VEGF showed an increasing trend from 15 minutes up to 24 hours. CaCl_2_ induced a progressive release of GFs from 15 minutes and increasing up to 24 hours. The method chosen to activate PRP influences both its physical form and the releasate in terms of GF amount and release kinetic.

## 1. Introduction

Tissue repair in musculoskeletal injuries is often a slow and sometimes incomplete process, with patient suffering pain and limited function, and therefore it is accompanied by high costs to society, in terms of both money spent on healthcare and also loss of work. Thus, many efforts have been made in order to investigate new approaches to increase the regenerative potential and favour tissue healing. Since several studies have underlined the role of growth factors (GFs) in the regulation of normal tissue structure and the reaction to tissue damage, their use is thought to be useful in clinical practice to promote rapid healing with high quality tissue and allow an early and safe return to unrestricted activity [1].

Platelets constitute a reservoir of critical GFs and cytokines which may govern and regulate the tissue healing process. The bioactive molecules secreted by platelet *α*-granules are involved in several cellular activities such as stem cell trafficking, proliferation, and differentiation, with a complex effect on pro/anti-inflammatory and anabolic/catabolic processes [2]. Moreover, with respect to purified individual GFs, platelets have the theoretical advantage of containing various bioactive molecules with a natural balance of anabolic and catabolic functions, possibly optimizing the tissue environment and favouring the healing process [2]. Based on this rationale, Platelet-Rich Plasma (PRP) is an easy, low-cost, and minimally invasive procedure to deliver high concentrations of autologous GFs and cytokines into injured tissues in physiological proportions. This blood-derived product, placed directly into the damaged tissue, either surgically or through injections, has been widely experimented in different fields of medicine [3–10].

However, despite the numerous benefits ascribed to PRP and the promising results reported for its therapeutic potential, the clinical outcomes are heterogeneous and sometimes contradictory. These controversial findings are due to both the different clinical protocols applied, making it difficult to compare results and draw conclusions about its real efficacy, and even more so the lack of standardization in PRP preparation procedures. This has led to the availability of a huge number of products differing in terms of cell types and quantity and therefore GF and cytokine content and release times. Among the several variables affecting PRP releasate, platelet activation is a crucial step that might influence the availability of bioactive molecules and therefore tissue healing [10, 11].

The term “activation” refers to 2 key processes that are initiated during PRP preparation: (1) degranulation of platelets to release GFs from *α*-granules and (2) fibrinogen cleavage to initiate matrix formation, a clotting process which allows the formation of a platelet gel, and therefore to confine the secretion of molecules to the chosen site [12]. An activation step before PRP administration is included in many of the protocols used, commonly by adding thrombin and/or calcium chloride (CaCl_2_), but some physicians prefer to inject PRP in its resting form, relying on the spontaneous platelet activation occurring after exposure to the native collagen present in the connective tissues [13]. Currently, there is a lack of evidence on the most suitable method for PRP activation, and the choice of strategy to activate it is mainly based on practical reasons rather than supported by studies on the effects of the different procedures on the final platelet releasate. The definition of the differences among activation methods might allow PRP preparations to be optimized, by identifying the most suitable strategy for each specific pathology, in order to obtain a customized PRP for the various clinical indications.

The aim of the present study is therefore to compare different strategies to activate PRP, by evaluating the content of both GFs and cytokines, as well as their release kinetics.

## 2. Materials and Methods

This study was approved by the local Ethics Committee and the Institutional Review Board, and each donor signed an informed written consent. PRP, Platelet-Poor Plasma (PPP), and autologous thrombin were obtained from ten voluntary healthy men (mean age ± SD: 31.4 ± 5.1 years) who underwent a blood sample collection of 150 mL. Subjects did not present with systemic disorders, smoking habit, infection, nonsteroidal anti-inflammatory drug use in the 5 days before blood donation, haemoglobin lower than 11 g/dL, or platelets lower than 150 × 10^3^/*μ*L. A code number was assigned to each sample to ensure the subject's anonymity.

### 2.1. Preparation and Activation of Blood Derivatives

PRP, PPP, and autologous thrombin were prepared by a whole blood separator (Angel, Cytomedix Inc., Gaithersburg, MD). For PRP preparation, 150 mL of venous blood was drawn from each donor and transferred into an Angel centrifuge chamber and centrifuged for 25 min at two different speeds: at 3500 rpm for the first 3 minutes and at 3000 rpm for the remaining time. Then, PRP was extracted from the buffy-coat into an empty sterile syringe. PPP was collected from another bag and transferred into a new syringe. Autologous thrombin was prepared starting from PPP according to the manufacturer's instructions.

### 2.2. Platelet Concentration and White Blood Cell Number

The platelet concentration and the white blood cell (WBC) number of PRP, PPP, and peripheral blood (PB) were analysed with an automated blood cell counter (COULTER LH 750 Haematology Analyzer Beckman Coulter SRL, Milan, Italy). Linearity was 5–1000 × 10^3^/*μ*L for platelet count and 0.1–100 × 10^3^/*μ*L for white blood cell count.

### 2.3. PRP Activation

Activation of PRP was performed by adding 10% of CaCl_2_ (final concentration 22.8 mM), 10% of autologous thrombin, 10% of a mixture of CaCl_2_ + thrombin, and 10% of collagen type I (final concentration 4 *μ*g) (Mascia Brunelli SpA, Milan). PRP without activation and PPP were used as control. Blood derivatives were incubated for 15 and 30 minutes and 1, 2, and 24 hours at 37°C. Then, samples were centrifuged at 2800 ×g for 15 minutes at 20°C, and the supernatants were collected and stored at −80°C until use.

### 2.4. Growth Factor Evaluation

PRP and PPP were evaluated for the release of VEGF, TGF-*β*1, PDGF-AB, IL-1*β*, and TNF-*α* (R&D Systems, Minneapolis, MN). Samples were thawed at 4°C and centrifuged before analysis; then they were assayed in duplicate and factors were evaluated using quantitative sandwich enzyme immunoassays following the manufacturer's instructions. Results were expressed as pg/mL.

### 2.5. Statistical Analysis

All continuous normally distributed data were expressed in terms of the mean and the standard deviation of the mean; the median was used for not normally distributed ones. The Kolmogorov Smirnov test was performed to test normality of continuous variables. The area under the curve of release at every time of measurement was calculated for each activation method to quantify the amount and kinetics of the released molecules. The Repeated Measures General Linear Model (GLM) with Sidak test for multiple comparisons was performed to assess the differences at different follow-up times in each activation method. The Repeated Measures GLM was also used to assess the influence of the different activation methods.

For all tests, *p* < 0.05 was considered significant.

All statistical analyses were performed using SPSS v. 19.0 (IBM Corp., Armonk, NY, USA).

## 3. Results

### 3.1. Platelet Concentration and White Blood Cell Number

The median number of platelets per cubic millimeter was 215.7 ± 76.3, 23.7 ± 13.6, and 974.7 ± 353.2 for PB, PP, and PRP, respectively. The median concentration of white blood cells per cubic millimeter was 5.4 ± 0.8, 0.1 ± 0.0, and 19.7 ± 6.4 for PB, PP, and PRP, respectively.

### 3.2. Clot Formation

**Figure 1:**
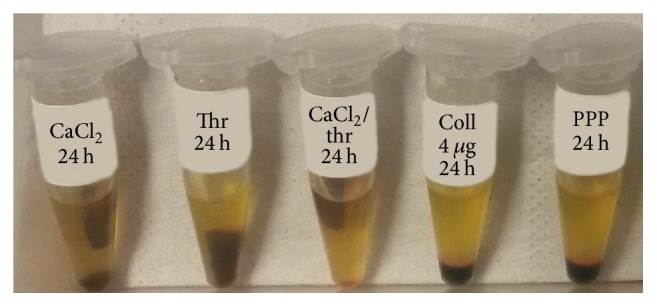
Clot formation at 24 h in the different activation groups.

CaCl_2_, thrombin, CaCL_2_/thrombin, and collagen type I induced a different platelet aggregation. In particular, PRP activated with CaCl_2_, thrombin, and CaCL_2_/thrombin formed clots detected in the 15-minute evaluation and persisting up to 24 hours (thrombin and CaCL_2_/thrombin already macroscopically stable at 15 minutes, CaCl_2_ starting at 15 and visually stabilized at 30 minutes), whereas in collagen-type-I-activated samples no clot formation was noticed for any of the time points evaluated (Figure 1).

### 3.3. Growth Factor and Cytokine Release

No detectable levels of IL-1*β* and TNF-*α* secretion were found in any of the samples at any of the experimental times evaluated.

Significantly lower amounts of GFs were detected in nonactivated PRP and PPP compared to the differently activated PRP (*p* < 0.05).

**Figure 2:**
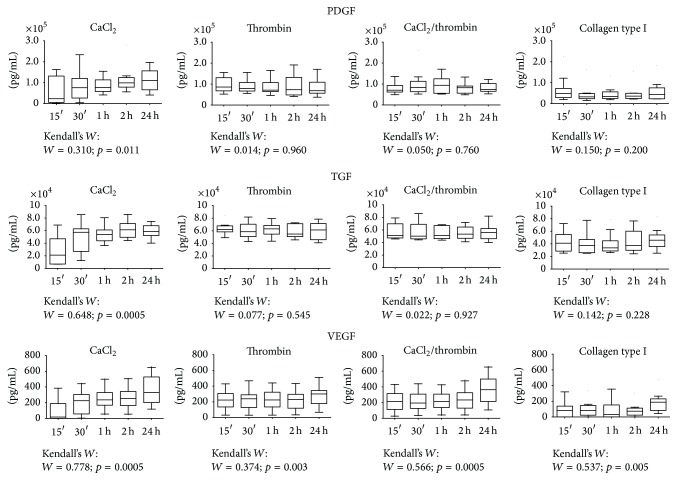
TGF, PDGF, and VEGF release kinetics according to the activation method.

The effects of the different activation methods on PRP GF release are shown in Figure 2 in detail.

#### 3.3.1. PDGF

At 15 and 30 minutes, thrombin and CaCl_2_/thrombin produced a significantly higher amount of PDGF with respect to that of CaCl_2_ (*p* < 0.05). After 1 h, CaCl_2_ and thrombin alone produced a similar amount of PDGF, whereas the combination of CaCl_2_/thrombin induced a significantly higher PDGF release with respect to that of CaCl_2_ (*p* < 0.05). Moreover, thrombin and CaCl_2_/thrombin showed greater amount of PDGF with respect to that of collagen type I (*p* < 0.05). At 2 hours CaCl_2_, thrombin, and CaCl_2_/thrombin produced similar levels of PDGF, all significantly higher compared to those of collagen type I. After 24 h, PDGF release from CaCl_2_ activated PRP was significantly higher with respect to that of thrombin (*p* < 0.05), and CaCl_2_ and CaCl_2_/thrombin induced more PDGF compared to that of collagen type I (*p* < 0.05) (Figure 2).

#### 3.3.2. TGF-*β*

At 15 minutes, thrombin and CaCl_2_/thrombin showed a greater amount of TGF-*β* with respect to that of CaCl_2_ and collagen type I (*p* < 0.05), whereas no significant difference was noted between CaCl_2_ and collagen type I. After 30 minutes, thrombin and CaCl_2_/thrombin produced a significantly higher amount of TGF-*β* with respect to collagen type I (*p* < 0.05). At 1 hour, thrombin induced a significantly higher TGF-*β* release with respect to that of CaCl_2_ (*p* < 0.05), while CaCl_2_, thrombin, and CaCl_2_/thrombin were higher with respect to that of collagen type I (*p* < 0.05). Significantly higher levels of TGF-*β* were observed for CaCl_2_, thrombin, and CaCl_2_/thrombin with respect to that of collagen type I at both 2 and 24 hours (*p* < 0.05) (Figure 2).

#### 3.3.3. VEGF

At 15 and 30 minutes, thrombin and CaCl_2_/thrombin produced a significantly higher amount of VEGF with respect to that of CaCl_2_ and collagen type I (*p* < 0.05). After 1 hour, CaCl_2_ and thrombin alone produced a similar amount of VEGF, whereas the combination of CaCl_2_/thrombin induced a significantly higher VEGF release with respect to that of CaCl_2_ (*p* < 0.05). Moreover, CaCl_2_, thrombin, and CaCl_2_/thrombin showed a greater amount of VEGF with respect to that of collagen type I (*p* < 0.05). At both 2 h and 24 hours CaCl_2_, thrombin, and CaCl_2_/thrombin produced significantly higher VEGF levels compared to those of collagen type I (*p* < 0.05). Finally, CaCl_2_/thrombin produced a significantly higher amount of VEGF with respect to that of thrombin at 24 hours (*p* < 0.05) (Figure 2).

### 3.4. Growth Factor Release Kinetics

The release pattern of PRP activated with CaCl_2_ was similar for all the GFs evaluated, with a significant and progressive release of GFs starting from 15 minutes and increasing up to 24 hours (*p* < 0.05) (Figure 2). Thrombin, CaCL_2_/thrombin, and collagen-type-I-activated PRP showed an immediate release of PDGF and TGF-*β*_1_ that remained stable over time (Figure 2); conversely, VEGF showed an increasing trend from 15 minutes up to 24 hours (*p* < 0.05).

## 4. Discussion

The main finding of our study is that the activation modality influences PRP clot formation, leading to differences in terms of both amount and release kinetics of platelet-derived GFs.

The most commonly used activation methods in the current clinical practice [14–16] were directly compared: CaCl_2_, autologous thrombin, their combination, and collagen type I to mimic the clinical conditions where their presence in the treated connective tissues should induce an “*in situ*” platelet activation. The latter is currently chosen for several PRP applications, since it is considered to be an easier and more effective strategy to deliver platelet bioactive molecules. However, our data showed that collagen type I does not lead to the same PRP releasate with respect to the other activators in terms of GFs released. In this study, we evaluated 3 GFs chosen among the most representative of PRP and involved in the wound healing cascade (TGF-*β*1, PDGF-AB, and VEGF) and 2 inflammatory mediators (IL-1*β* and TNF-*α*) to test whether the selected activators might induce an inflammatory component of PRP releasate. The* in vitro* results showed significantly lower quantities of TGF-*β*1, PDGF-AB, and VEGF when collagen type I was used in this experimental condition.

Collagen is a weak platelet activator, which results in a lower amount of GFs released with respect to the other activation methods. This is a key aspect to bear in mind, since GFs are potent molecules and even small variations might affect the results in the tissue healing process [17, 18]. In fact, although low concentrations may be not effective enough to elicit the desired effects, high GF concentrations may have inhibitory effects on cellular functions and the level of the healing response [19–21]. Besides, they can be associated with unresolved inflammation and fibrotic events [22], thus confirming the importance of obtaining a proper releasate, also by choosing a specific PRP activation method to stimulate the release of bioactive molecules according to the requirements of the targeted tissue.

Besides the overall higher amount of released GFs with the other activation strategies used in the clinical practice, the comparison of the amount of molecules detected at each time point underlined another key factor related to PRP activation: the different release kinetics. This is of major importance and may also affect the treatment outcome. In fact, a rapid activation has been associated with a decrease in the total amount of GFs available at the tissue site over time [23]. GFs have a short half-life (from minutes to hours) and, if they are not immediately used upon release from platelets, they might be degraded before additional tissue receptors become available [15, 24]. From a clinical point of view, this aspect may be one of the causes related to the poor results sometimes reported by using PRP in musculoskeletal tissue regeneration [23]. Conversely, some other applications may benefit from a less sustained release, with the final results determined by the burst of bioactive molecules released [25, 26].

The study results highlighted that thrombin alone and in combination with CaCl_2_ and collagen type I (even if at lower level in this case) presented similar kinetics, stimulating a rapid release of GFs that remains stable up to 24 h. Similarly, comparing the PRP releasate induced by thrombin or collagen type I, Fufa et al. [14] observed that both activation methods stimulate immediate initial release sustained over 10 days from a PRP clot.

Conversely, CaCl_2_ showed a gradual release over time, with a lower initial level followed by a progressively increasing amount of GFs released, reaching similar or even higher levels at the 24-hour evaluation.

Another important aspect for the clinical application of blood derivatives is their physical form, which may range from liquid to solid gel allowing both surgical augmentations as well as minimally invasive injective PRP delivery. Concerning this, the study underlined how different activators influence platelet aggregation. In particular, the use of CaCl_2_ induced clot formation within 30 minutes of its addition, whereas thrombin and CaCl_2_/thrombin caused a more rapid clot formation, which was already detectable at the 15-minute evaluation. Interestingly, collagen-type-I-activated platelet concentrates exhibited far less aggregation, with no visible clots up to 24 hours. This result is partially in contrast to that obtained by Fufa et al. [14], who observed that PRP activation with collagen led to clot formation, albeit far less retracted than that observed with thrombin activation. This discrepancy may be due to the different experimental conditions, such as the type of collagen and the different procedures used to prepare PRP. PRP may present a wide range in terms of type and quantity of cells and molecules such as fibrinogen, which may explain the different propensity to form clots. The state of the platelet concentrate may be as important as the released molecules for treatment success. In fact, although the lack of a clot might not be a problem in the treatment of osteoarthritis, where a liquid PRP allows all articular tissues to be targeted without the risk of dispersion from the closed joint cavity [5], the liquid form may be unsuitable for other applications. This appears to be clear for surgical augmentations, where PRP is used to entrap cells or even sutured to the lesion site [23], but may also apply for less invasive injective approaches. This may be the case of intratendinous injections. Once delivered inside the tendon in the liquid form, the timely gelification process may allow the concentrate to remain in the injected area, thus allowing GF secretion in the treatment site. Conversely, the persistent liquid state may increase the risk of leakage and PRP dispersion, favoured by the contraction of the musculotendinous unit that might squeeze liquid PRP away from the injection site, thus reducing or even impairing its potentially positive effects [13]. A direct PRP activation* in situ* is an interesting approach that might overcome some of the shortcomings related to the use of thrombin, resulting in the risk of potentially life-threatening coagulopathies [27, 28], and CaCl_2_, sometimes associated with burning sensation due to low pH, as reported by DeLong et al. [23]. However, the results obtained in this study with collagen-mediated activation cast doubts on this method for PRP application in the clinical practice. Further studies should focus not only on the distribution of the injected PRP in the treated area, but also on its persistence over time and on the consequent effects on the final outcome for each specific application [29].

Finally, besides the differences in clot formation and GFs amount and release kinetics, another interesting finding that emerged from the study analysis regards the lack of influence of the activation methods on the inflammatory molecules in the releasate. The PRP used in this experimental setting is a leukocyte-rich PRP, which is currently being debated for the potentially deleterious effects of proteases and reactive oxygen species released by the white blood cell component. In fact, whereas some authors consider leukocytes to be a beneficial source of cytokines and enzymes that may be important for the prevention of infection, others attribute better results to formulations with leukocyte depletion [30–32].

The study results showed that, even in this leukocyte-rich PRP, none of the selected activators was able to induce an inflammatory releasate, as demonstrated by the lack of IL-1*β* and TNF-*α* secretion at all the experimental times evaluated. Future studies should investigate if the same findings will be confirmed also in an inflammatory environment better reproducing PRP use in the damaged tissues of the clinical setting.

This study has some limitations that need to be discussed. In fact, today little is known about the concentration of calcium, thrombin, or collagen needed to trigger the optimal release of GFs, and different concentrations may lead to different results. For example, it has been reported that high concentrations of calcium and thrombin trigger an immediate and significant increase in TGF-*β*1 and PDGF concentrations, which remained generally constant over a 6-day period, whereas lower concentrations tend to reduce and delay GF release [26]. However, the selected concentrations derive from the clinical practice; thus, the study findings still reflect the current PRP applications. Nonetheless, the activator concentration should be the focus of further specifically designed studies, being a key aspect to determine the final properties of the platelet concentrates. Moreover, these findings give only general indications that could be useful for future PRP application in musculoskeletal tissues, and further studies are needed to investigate the effects of different PRP activation methods on cell cultures.

The results of this study confirm the importance of the method chosen to activate PRP, by determining both its physical form and the amount and release kinetics of GFs. It is not only the presence of GFs that dictates the level of healing response, but also the ability of targeting the treatment area, thus modulating cells with an appropriate dosage and in a timely manner [33]. Thus, PRP activation strategies should be selected not only based on procedure type (open versus arthroscopic), but also according to the desired biological effects in the targeted tissue. Future studies should aim at further investigating the effect of the different activation strategy on platelet concentrates according to the lesion target, in order to optimize the* in vivo* effect of the released bioactive molecules and therefore increase PRP healing potential.
